# Machine learning identification and immune infiltration of disulfidptosis‐related Alzheimer's disease molecular subtypes

**DOI:** 10.1002/iid3.1037

**Published:** 2023-10-11

**Authors:** Yidong Zhu, Lingyue Kong, Tianxiong Han, Qiongzhi Yan, Jun Liu

**Affiliations:** ^1^ Department of Traditional Chinese Medicine, Shanghai Tenth People's Hospital Tongji University School of Medicine Shanghai China

**Keywords:** Alzheimer's disease, disulfidptosis, gene model, immunity, machine learning, molecular subtypes

## Abstract

**Background:**

Alzheimer's disease (AD) is a common neurodegenerative disorder. Disulfidptosis is a newly discovered form of programmed cell death that holds promise as a therapeutic strategy for various disorders. However, the functional roles of disulfidptosis‐related genes (DRGs) in AD remain unknown.

**Methods:**

Microarray data and clinical information from patients with AD and healthy controls were downloaded from the Gene Expression Omnibus database. A thorough examination of DRG expression and immune characteristics in both groups was performed. Based on the identified DRGs, we performed an unsupervised clustering analysis to categorize the AD samples into various disulfidptosis‐related molecular clusters. Weighted gene co‐expression network analysis was performed to select hub genes specific to disulfidptosis‐related AD clusters. The performances of various machine learning models were compared to determine the optimal predictive model. The predictive ability of the optimal model was assessed using nomogram analysis and five external datasets.

**Results:**

Eight DRGs showed differential expression between the AD and control samples. Two different molecular clusters were identified. The immune cell infiltration analysis revealed distinct differences in the immune microenvironment of the two clusters. The support vector machine model showed the highest performance, and a panel of five signature genes was identified, which showed excellent performance on the external validation datasets. The nomogram analysis also showed high accuracy in predicting AD.

**Conclusion:**

We identified disulfidptosis‐related molecular clusters in AD and established a novel risk model to assess the likelihood of developing AD. These findings revealed a complex association between disulfidptosis and AD, which may aid in identifying potential therapeutic targets for this debilitating disorder.

## Graphical abstract

Our study identified molecular clusters related to disulfidptosis in AD and established a novel model to evaluate the risk of AD development.

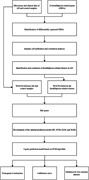

## 1 INTRODUCTION

Alzheimer's disease (AD) is the foremost cause of dementia and is becoming one of the most lethal and burdensome diseases.^1^, ^2^ The most common clinical manifestation of AD is slowly progressing amnesia, which reflects the pathology of early neurofibrillary tangles in the medial temporal lobe, eventually evolving into multidomain dementia dominated by amnestic predominance.^3^ Genetic factors account for 60%–80% of the risk of AD, and more than 40 genetic risk loci associated with AD have been identified, among which apolipoprotein E alleles exhibit the strongest correlation with this disease.^1^, ^4^ Only a few medical treatments have been approved for AD, and these mainly focus on managing symptoms rather than altering the disease course.^5^, ^6^ Although research on potential disease‐modifying therapies has mainly focused on detecting the disease clinically, there is evidence that pathology related to AD begins several years before this stage.^7^ In the preclinical phase, pharmacological therapy may be beneficial before the onset of neurodegenerative processes. However, growing evidence has shown that AD is a heterogeneous disease caused by multiple pathophysiological mechanisms, and predicting its progression is challenging.^8^ Therefore, there is no “one size fits all” intervention, and individualized treatment choices are recommended.^9^ Taken together, it is crucial to identify dependable diagnostic markers for early AD detection and devise novel molecular stratification methods aimed at directing precision medicine.

The abnormal accumulation of disulfides in cells causes disulfide stress, which may result in high levels of cellular toxicity.^10^, ^11^ Recently, a disulfide‐triggered modality of regulated cell death was reported, which was termed “disulfidptosis.”^12^ Different from other programmed cell death processes, disulfidptosis is mediated by the sensitivity of the actin cytoskeleton to disulfide stress,^12^ which suggests a promising strategy for treating various diseases. The functional roles of programmed cell death‐related genes in AD development have been previously reported. For instance, genes related to cuproptosis,^13^, ^14^ pyroptosis,^15^ and ferroptosis^16^, ^17^ have been used to construct prediction models for AD. However, as the underlying mechanism of disulfidptosis has been discovered, the potential link between disulfidptosis‐related genes (DRGs) and AD remains unclear. Currently, studies on disulfidptosis have mainly focused on cancers.^18^, ^19^, ^20^ A recent study has reported that the dysregulation of actin cytoskeletal dynamics is associated with the pathology of AD.^21^ The accumulation of disulfide bonds and the mechanism of disulfidptosis may damage the actin cytoskeleton, suggesting a potential relationship between disulfidptosis and AD.

In recent years, the field of machine learning has witnessed widespread application in predicting biomarkers and offering fresh insights into the pathogenesis of diseases owing to its excellent performance in clinical diagnosis.^22^, ^23^, ^24^, ^25^ Several studies have used machine learning to classify individuals at risk of progressing to AD.^26^, ^27^, ^28^, ^29^, ^30^ Therefore, in this study, we aimed to identify molecular clusters related to disulfidptosis and establish a novel risk model to assess the likelihood of developing AD based on machine learning. With this objective, we investigated the expression patterns of DRGs in AD and control samples. We then categorized patients with AD into two disulfidptosis‐related clusters based on DRG expression patterns and evaluated immune cell differences. By performing weighted gene co‐expression network analysis (WGCNA), we identified hub genes specific to the disulfidptosis‐related AD clusters. Subsequently, a prediction model was formulated using multiple machine‐learning algorithms. The performance of the optimal model was validated using nomogram analysis and five external datasets. These results provide valuable insights into the diagnosis and molecular stratification of AD.

## 2 MATERIALS AND METHODS

### 2.1 Data collection

Microarray data and clinical characteristics of both AD and control samples were obtained from the Gene Expression Omnibus (GEO) database (https://www.ncbi.nlm.nih.gov/geo/). Raw data were normalized to eliminate batch effects. We used the GSE33000 dataset as a training cohort. Additionally, five different validation cohorts, namely GSE5281, GSE36980, GSE48350, GSE122063, and GSE132903, were selected to verify the accuracy of our results. Table 1 presents the clinical characteristics of these datasets.

**Table 1:** Characteristics of the studied datasets.

GEO series	Control samples	AD samples	Age ≤ 80	Age > 80	Male	Female	Data type
GSE5281	74	87	96	65	103	58	Validation cohort
GSE33000	157	310	298	169	258	209	Training cohort
GSE48350	173	80	142	111	124	129	Validation cohort
GSE122063	44	56	36	64	32	68	Validation cohort
GSE132903	98	97	49	146	99	96	Validation cohort
GSE36980	47	33	29	51	37	43	Validation cohort

Abbreviation: AD, Alzheimer's disease.

### 2.2 Identification of differentially expressed DRGs

We obtained 10 DRGs for this study from the literature (Supporting Information: Table S1).^12^ The expression data of DRGs in the AD and control samples from the training set were extracted, and differential expression analysis was performed using the “limma” package. DRGs with *p* < 0.05 were considered differentially expressed.

### 2.3 Immune cell infiltration and correlation analyses

The relative abundance of 22 types of infiltrating immune cells in each sample was calculated using the CIBERSORT algorithm. We compared the enrichment levels of infiltrating immune cells between the AD and control samples to investigate the potential association between AD and immunity. We further assessed the correlation between differentially expressed DRGs and infiltrating immune cells by performing Spearman's correlation analysis. Data analysis and visualization were performed using the “e1071,” “reshape2,” “ggpubr,” “tidyverse,” and “ggplot2” packages.

### 2.4 Identification and evaluation of disulfidptosis‐related clusters in AD

Based on the expression of the identified differentially expressed DRGs, an unsupervised clustering analysis was performed to categorize the AD samples in the training cohort into various clusters using the “ConsensusClusterPlus” package. We comprehensively evaluated the optimal number of clusters by analyzing the consensus matrixs, cumulative distribution function (CDF) curves, and consensus scores. Principal component analysis (PCA) was performed to depict the distribution of the identified clusters visually. A differential expression analysis was performed to evaluate differences in the expression of DRGs among the various clusters. Additionally, an immune cell infiltration analysis was performed to examine the characteristics of immune cell infiltration among the different clusters. Moreover, gene set variation analysis (GSVA) was performed to elucidate the differentially expressed pathways and biological mechanisms among the different disulfidptosis‐related clusters. Statistical analyses and data visualization were performed using various packages, including “pheatmap,” “reshape2,” “ggpubr,” “ggplot2,” “GSEABase,” and “GSVA.”

### 2.5 WGCNA

Genes that are commonly arranged in a co‐expression network where they frequently connect with other genes to occupy a core position in modules exhibiting high modular identity are called hub genes.^31^ WGCNA is a systematic molecular biology method that identifies correlation patterns among genes in microarray samples and pinpoints hub genes without subjectivity.^32^ Herein, we used the WGCNA approach on the training cohort and disulfidptosis‐related clusters to identify hub genes between the AD and control samples as well as between clusters 1 and 2. A scale‐free topology model was used to determine the optimal soft threshold by integrating goodness of fit with mean connectivity. Subsequently, multiple modules were identified in an unsupervised manner and their adjacencies and similarities were estimated using a topological overlap measure and average hierarchical clustering. The topologically similar modules were combined into a new cluster. Pearson's correlation analysis was performed to examine the associations between the module genes and clinical features, and the highest correlating module was selected. The module genes were further evaluated based on module membership (MM) and gene significance (GS). Candidate genes meeting the criteria of MM > 0.6 and GS > 0.5 between the AD and control samples and between clusters 1 and 2 were identified. Finally, hub genes were identified as those that overlapped between the candidate genes from both sets. Data analysis and visualization were performed using the “limma,” “WGCNA,” and “VennDiagram” packages.

### 2.6 Development of the optimal prediction model

Multiple machine‐learning algorithms were applied to develop a prediction model based on the identified hub genes. Random forest (RF) is a regression tree approach that uses predictor randomization and bootstrap aggregation to achieve a high degree of predictive accuracy.^33^ The support vector machine (SVM) algorithm can predict labels from one or more feature vectors by creating a decision boundary between two categories.^34^ The generalized linear model (GLM) is an extension of the classic linear model that has been widely used in statistics for parameter estimation.^35^ The extreme gradient boosting (XGB) algorithm has certain algorithmic optimizations and important features.^36^ Corresponding prediction models were constructed using the aforementioned algorithms. Subsequently, the feature importance and residual distributions of the models were analyzed. Receiver operating characteristic (ROC) curves were used to predict the specificity and accuracy of these models for AD diagnosis. Combined with the above predictive performance, an optimal machine learning model was obtained, and the top five feature variables in the model were identified as the optimal panel of signature genes. The analysis results were visualized using the “caret,” “DALEX,” “ggplot2,” “randomForest,” “kernlab,” “pROC,” and “xgboost” packages.

### 2.7 Nomogram construction

A nomogram was constructed based on the model to forecast disease risk. Nomogram analysis was performed to assess the predictive ability of the model. The results were visualized using the “rms” and “rmda” packages.

### 2.8 Validation of the gene prediction model

The ability of the model to distinguish between patients with AD and controls was assessed in five validation cohorts: GSE5281, GSE36980, GSE48350, GSE122063, and GSE132903 by performing ROC curve analysis. The “pROC” package was used to visualize the results.

### 2.9 Statistical analyses

Data were analyzed using the R software (version 4.1.3). Student's *t* test was used to detect the significance of differences between the AD and control samples. Two‐sided *p* < 0.05 was considered statistically significant.

## 3 RESULTS

### 3.1 Identification of differentially expressed DRGs

**Figure 1:**
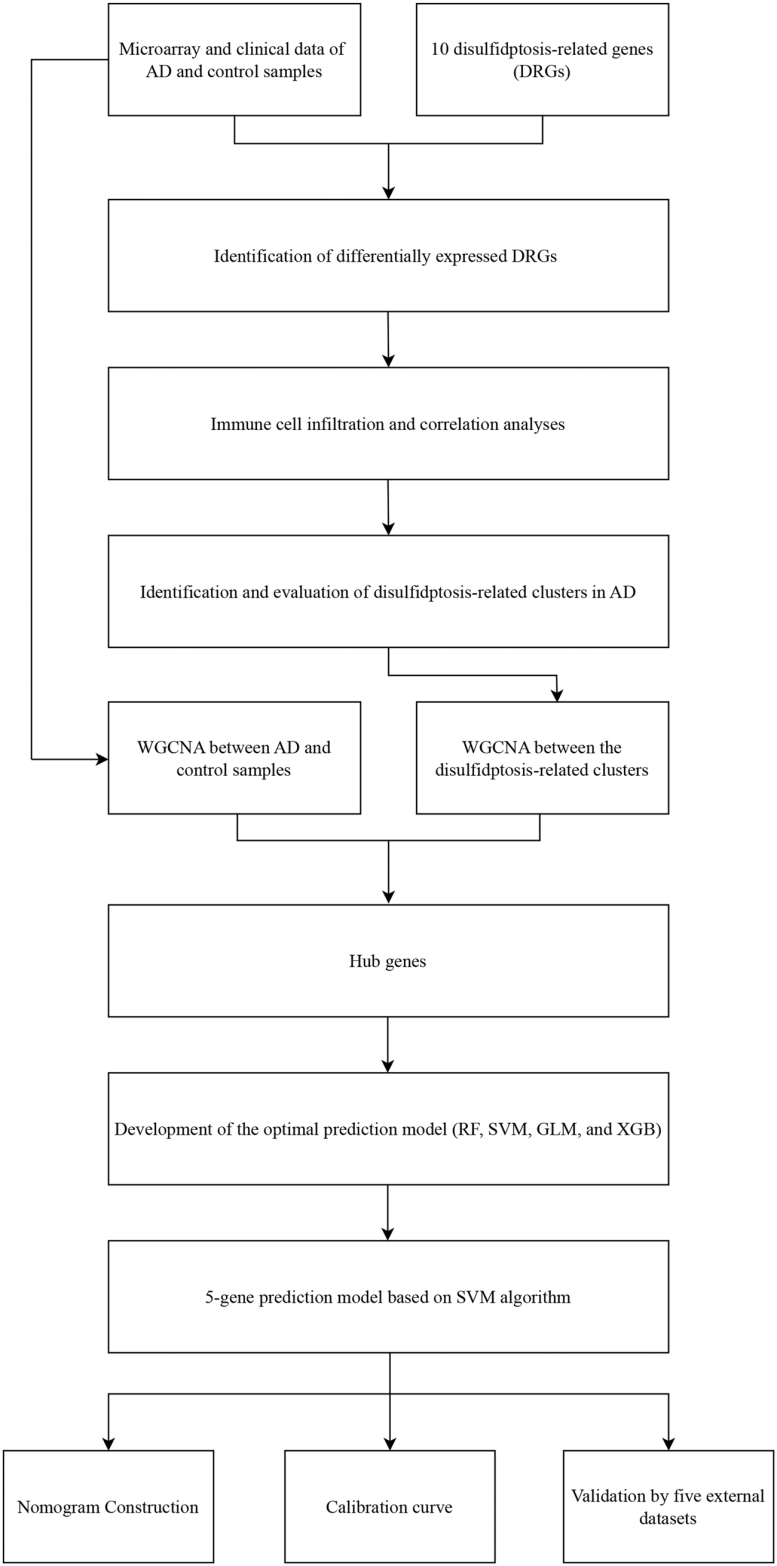
Flow diagram of the study. AD, Alzheimer's disease; DRG, disulfidptosis‐related gene; GLM, generalized linear model; RF, random forest; SVM, support vector machine; WGCNA, weighted gene co‐expression network analysis; XGB, extreme gradient boosting.

**Figure 2:**
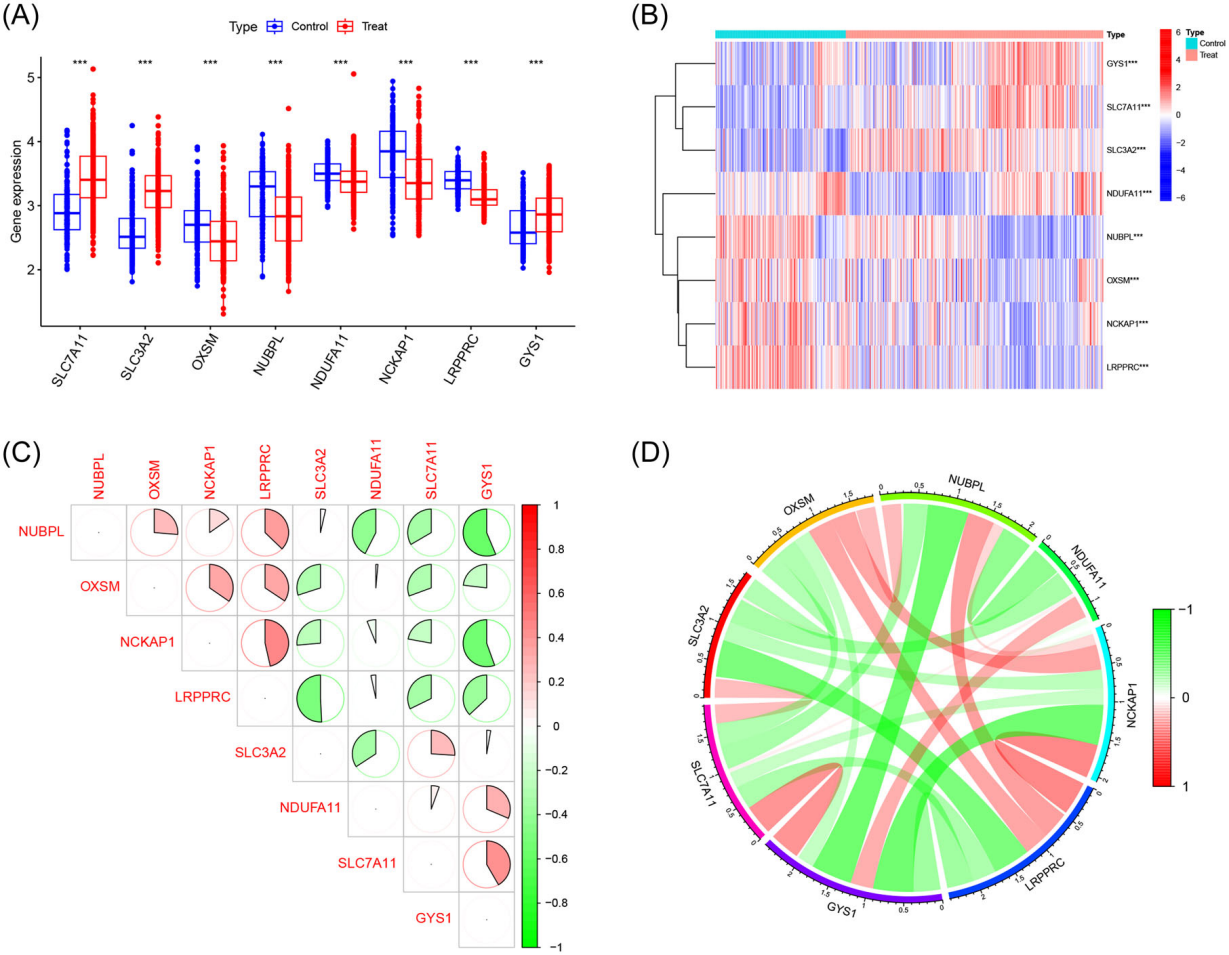
Identification of differentially expressed disulfidptosis‐related genes (DRGs). (A) Boxplot of differentially expressed DRGs between Alzheimer's disease (AD) and control samples; (B) heatmap of differentially expressed DRGs between AD and control samples; (C) correlation plot of differentially expressed DRGs; (D) gene relationship network diagram of differentially expressed DRGs.

Figure 1 shows a flowchart explaining the identification and immune characteristics of disulfidptosis‐related molecular clusters as well as the construction and validation of the predictive model for AD. A total of eight DRGs were differentially expressed between the AD and control samples (Figure 2A,B). *SLC7A11*, *SLC3A2*, and *GYS1* were upregulated, whereas *OXSM*, *NUBPL*, *NDUFA11*, *NCKAP1*, and *LRPPRC* were downregulated in the AD samples. Furthermore, the identified genes exhibited strong synergistic or antagonistic effects, and the interactions and interrelationships between these genes were visualized (Figure 2C,D).

### 3.2 Immune cell infiltration and correlation analyses

**Figure 3:**
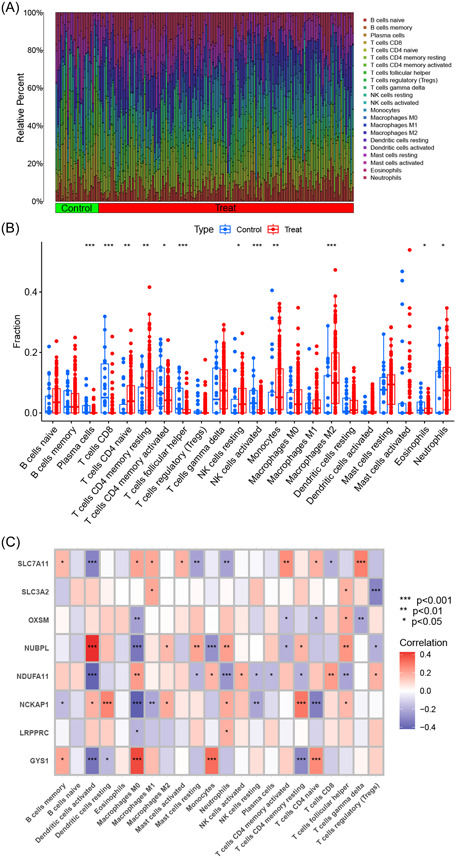
Immune cell infiltration and correlation analyses. (A) Relative abundance of 22 types of infiltrating immune cells in Alzheimer's disease (AD) and control samples. (B) Boxplot of immune‐related cells between AD and control samples. (C) Heatmap of correlations between differentially expressed disulfidptosis‐related genes and immune‐related cells.

The CIBERSORT algorithm was used to measure the relative abundance of 22 types of infiltrating immune cells in both AD and control samples (Figure 3A). Subsequently, immune cell infiltration analysis showed significant differences in 12 of 22 types of immune cells between the AD and control samples (Figure 3B), implying a potential role of immunological dysfunction in the pathogenesis and progression of AD. There was a significant correlation between differentially expressed DRGs and infiltrating immune cells (Figure 3C), suggesting that these genes may exert a profound effect on the immune infiltration status of patients with AD.

### 3.3 Identification and evaluation of disulfidptosis‐related clusters in AD

**Figure 4:**
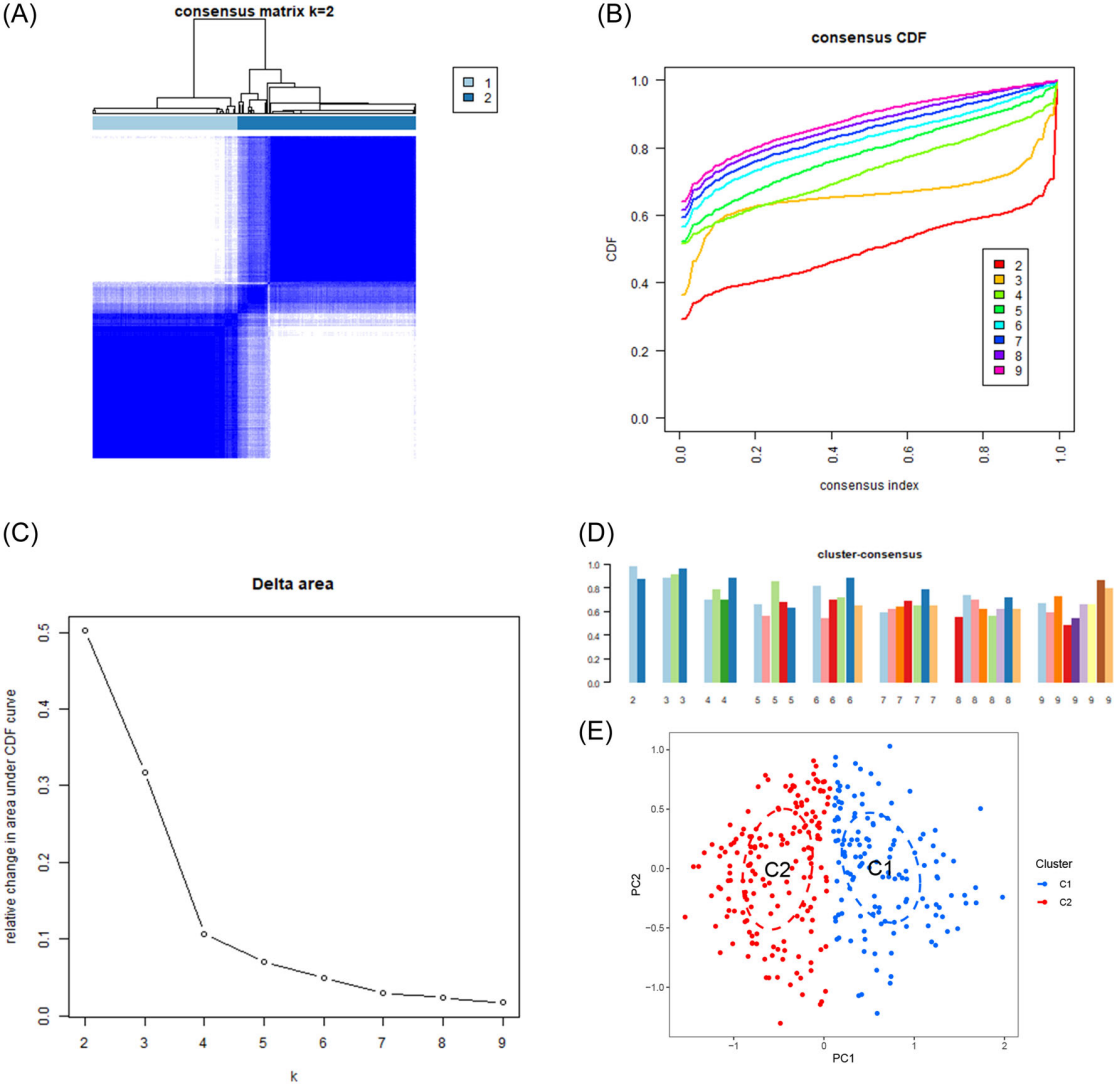
Identification of disulfidptosis‐related clusters in Alzheimer's disease. (A) Consensus clustering matrix for *k* = 2. (B–D) Representative cumulative distribution function curves. (E) Principal component analysis distribution of disulfidptosis‐related clusters.

An unsupervised clustering analysis was performed to classify the AD samples in the training cohort based on the identified differentially expressed DRGs, leading to the establishment of disulfidptosis‐related clusters for AD. The cluster matrix was the most consistent when *k* = 2, as confirmed by the consistent CDF curves and high consensus scores for each subtype (Figure 4A–D). Therefore, we identified two optimal clusters: cluster 1 (*n* = 139) and cluster 2 (*n* = 171). Additionally, PCA showed a clear differentiation between the previously mentioned clusters, suggesting the efficacy of unsupervised clustering for the AD samples (Figure 4E).

**Figure 5:**
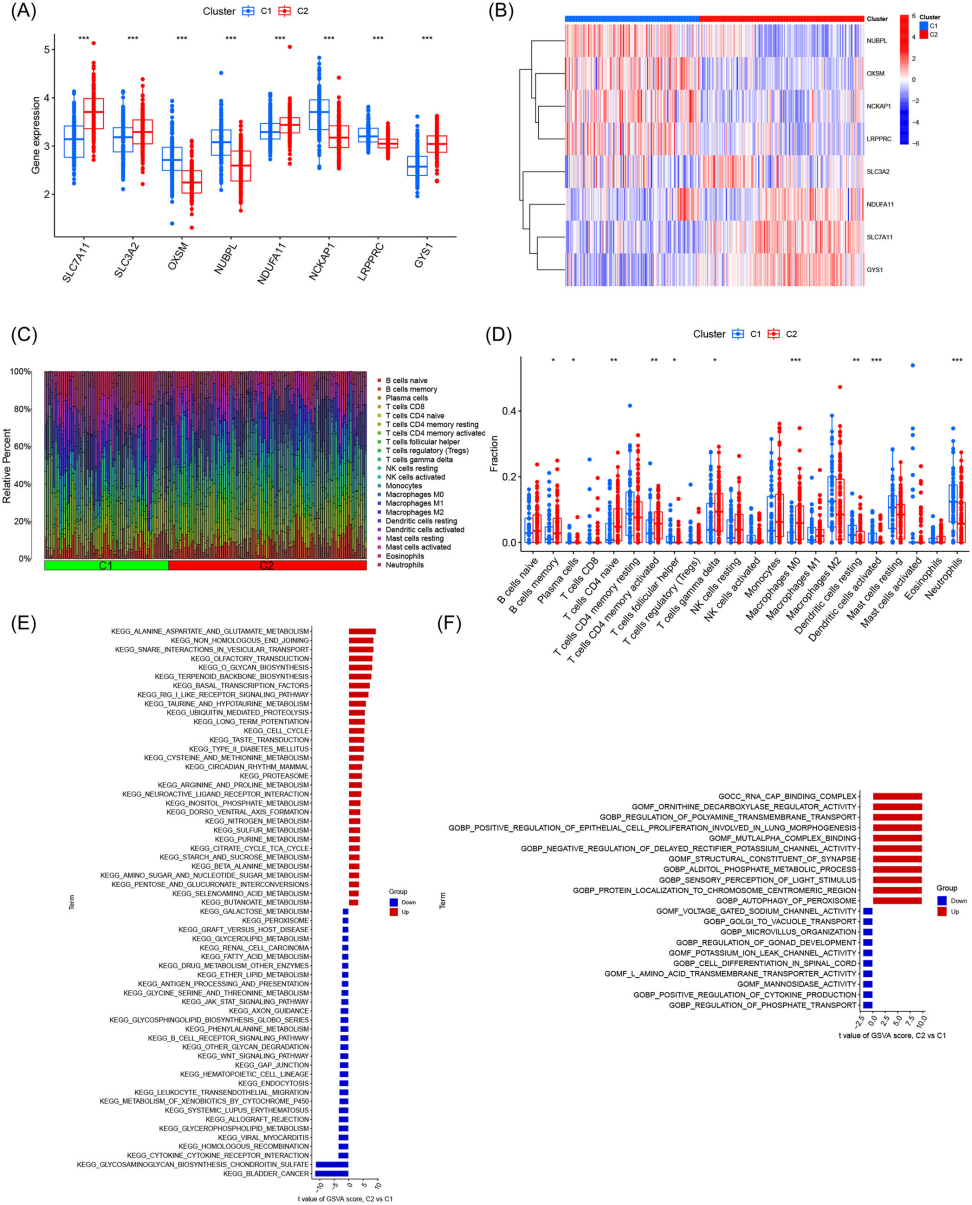
Evaluation of disulfidptosis‐related clusters in Alzheimer's disease. (A) Boxplot of differentially expressed disulfidptosis‐related genes (DRGs) between disulfidptosis‐related clusters; (B) heatmap of differentially expressed DRGs between disulfidptosis‐related clusters; (C) relative abundance of 22 types of infiltrating immune cells in disulfidptosis‐related clusters; (D) boxplot of immune‐related cells between disulfidptosis‐related clusters; (E) gene set variation analysis (GSVA) on the differentially expressed pathways among disulfidptosis‐related clusters; (F) GSVA on the differentially expressed biological mechanisms among disulfidptosis‐related clusters.

To investigate the molecular characteristics associated with disulfidptosis‐related clusters, we evaluated the expression of the eight DRGs in clusters 1 and 2. Cluster 1 showed the upregulated expression of *OXSM*, *NUBPL*, *NCKAP1*, and *LRPPRC*, whereas cluster 2 showed the upregulated expression of *SLC7A11*, *SLC3A2*, *NDUFA11*, and *GYS1* (Figure 5A,B). Additionally, the immune cell infiltration analysis revealed distinct differences in the immune microenvironment of the two clusters related to disulfidptosis (Figure 5C,D). Moreover, the GSVA revealed that cluster 1 was significantly associated with immune‐related pathways, including cytokine–cytokine receptor interaction, leukocyte transendothelial migration, and B‐cell receptor signaling pathway. In contrast, cluster 2 was mainly related to metabolic pathways such as alanine, aspartate, and glutamate metabolism, taurine and hypotaurine metabolism, and cysteine and methionine metabolism (Figure 5E). Additionally, the GSVA demonstrated that cluster 1 exhibited significant associations with regulation of phosphate transport, positive regulation of cytokine production, and mannosidase activity. On the other hand, cluster 2 displayed predominant connections with RNA cap‐binding complex, ornithine decarboxylase regulator activity, and polyamine transmembrane transport regulation (Figure 5F).

### 3.4 WGCNA

WGCNA was first performed on the training cohort to screen for hub genes related to AD and control samples. A soft threshold of 20 was determined, and the constructed network closely resembled a real biological network state, as evidenced by its adherence to a power‐law distribution (Supporting Infomation: Figure S1A). Hierarchical clustering analysis was performed, and the resulting clustering segments were merged to obtain seven modules (Supporting Infomation: Figure S1B,C). Among them, the blue module showed the strongest correlation between the AD and control samples (cor = .7, *p* = 5e^−70^; Supporting Infomation: Figure S1D). Additionally, we established a strong correlation between GS and MM (cor = .91, *p* = 3.4e^−169^). Based on the predetermined criteria, we identified 703 AD‐related genes in the blue module for subsequent analyses (Supporting Infomation: Figure S1E).

We then repeated the WGCNA approach on the disulfidptosis‐related clusters to identify hub genes associated with clusters 1 and 2 using a soft threshold of 5 (Supporting Infomation: Figure S2A). Hierarchical clustering analysis was performed, and the resulting clustering segments were merged to obtain nine modules (Supporting Infomation: Figure S2B,C). Among them, the blue module showed the strongest correlation between clusters 1 and 2 (cor = .71, *p* = 1e^−48^; Supporting Infomation: Figure S2D), and a close correlation between GS and MM (cor = .91, *p* = 2.3e^−147^) was also established. Using the predetermined criteria, 148 cluster‐specific genes were identified in the blue module (Supporting Infomation: Figure S2E). Finally, 58 overlapping genes were identified as hub genes specific to the disulfidptosis‐related AD clusters, based on the intersection of the 703 AD‐related genes from the training cohort and the 148 cluster‐specific genes from the disulfidptosis‐related clusters (Supporting Infomation: Figure S2F).

### 3.5 Development of the optimal prediction model

**Figure 6:**
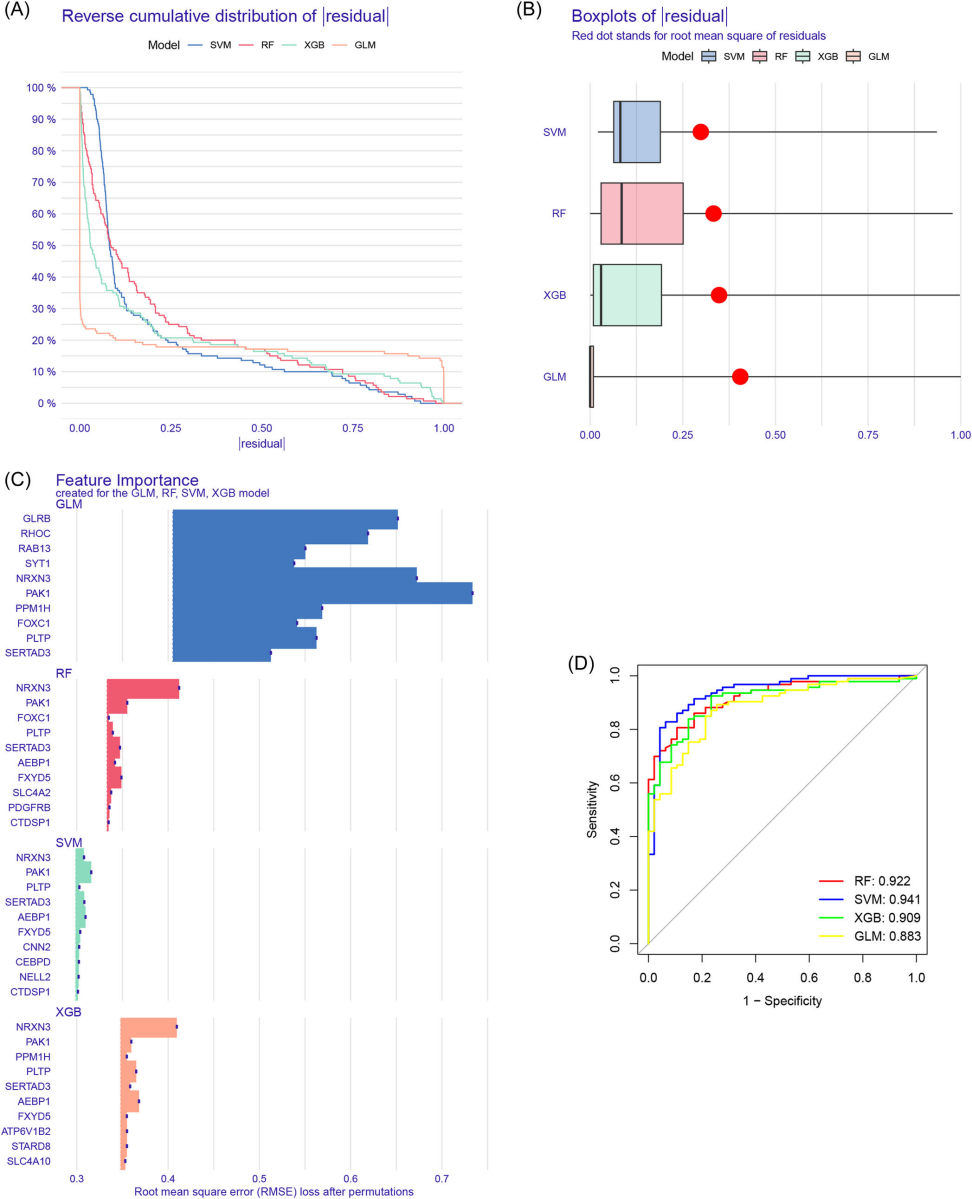
Development of the optimal prediction model. (A) Cumulative residual distribution of each machine‐learning model; (B) residuals of each machine‐learning model; (C) important feature variables of each machine‐learning model; (D) receiver operating characteristic curves of machine‐learning models in the training cohort.

Based on the identified 58 hub genes, multiple machine‐learning models were constructed according to the corresponding algorithms. Among them, the prediction model established by SVM exhibited the relatively lowest residuals (Figure 6A,B). Next, the top 10 important feature variables of each model were ranked according to the root mean square error (Figure 6C). Furthermore, the SVM model showed the highest area under the curve (AUC) value (RF, AUC = 0.922; SVM, AUC = 0.941; XGB, AUC = 0.909; GLM, AUC = 0.883, Figure 6D). Finally, combined with the above predictive performance, the prediction model established by the SVM algorithm exhibited superior diagnostic value for AD compared to the other algorithms used in this study. The following top five important feature variables in the SVM model were identified as the optimal panel of signature genes: *FXYD5*, *NRXN3*, *SERTAD3*, *AEBP1*, and *PAK1*.

### 3.6 Nomogram construction

**Figure 7:**
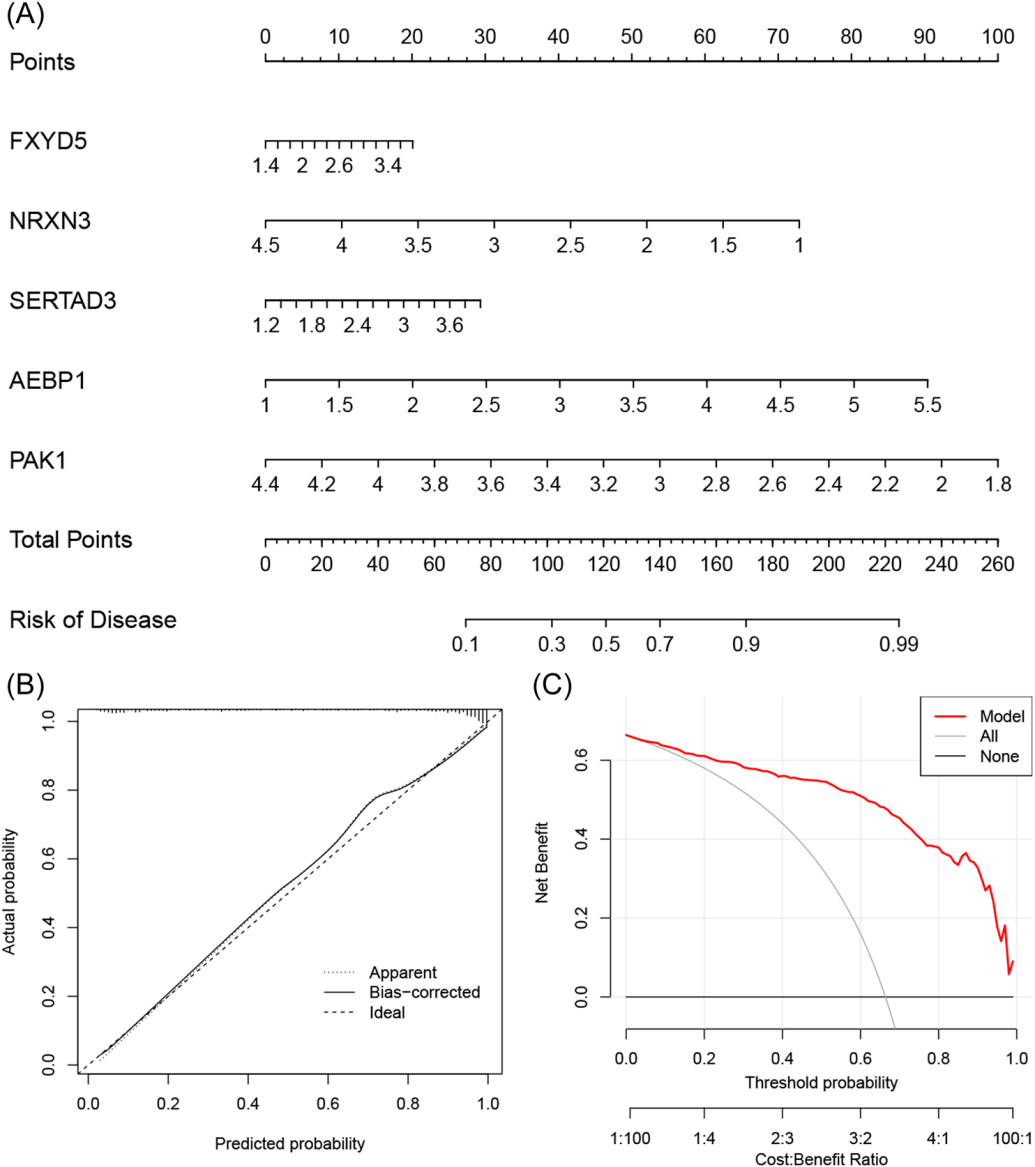
Nomogram construction. (A) Nomogram for predicting Alzheimer's disease risk; (B) calibration curve; (C) decision curve analysis.

A nomogram was constructed to predict the risk of developing AD (Figure 7A). The calibration curve indicated optimal concordance between the practical observation and the predicted risk probability (Figure 7B). The decision curve analysis showed that the constructed nomogram achieved a satisfactory benefit for clinical decision‐making (Figure 7C). These results indicate the good performance of the nomogram model in AD diagnosis.

### 3.7 Validation of the prediction model

**Figure 8:**
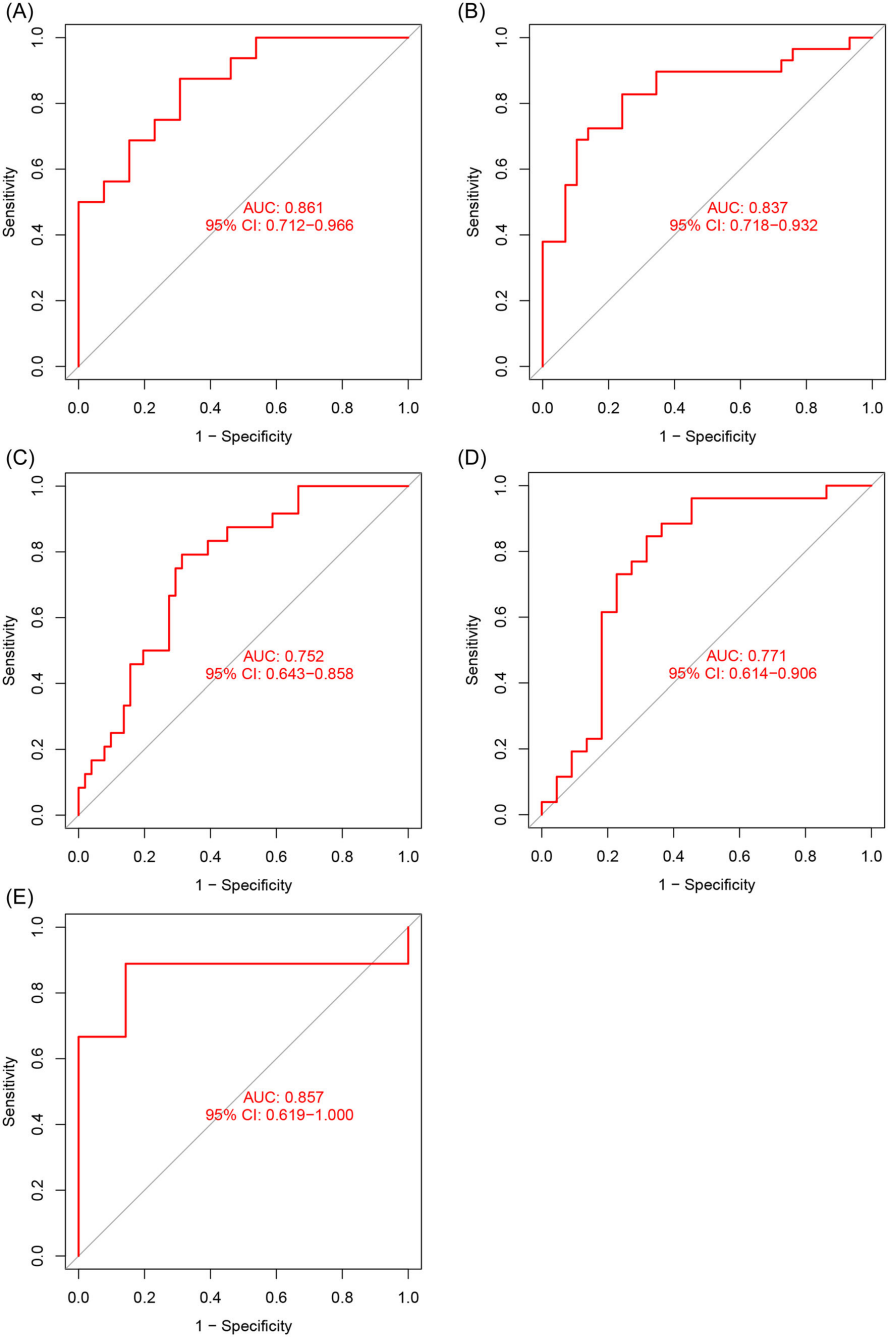
Validation of the gene prediction model. Receiver operating characteristic curves of the prediction model in the GSE122063 (A), GSE132903 (B), GSE48350 (C), GSE5281 (D), and GSE36980 (E) datasets. AUC, area under the curve; CI, confidence interval.

The five‐gene model was validated using five external datasets containing AD and control samples. The ROC curves demonstrated satisfactory model performance, with AUC values of 0.861, 0.837, 0.752, 0.771, and 0.857 for the GSE122063, GSE132903, GSE48350, GSE5281, and GSE36980 datasets, respectively (Figure 8A–E). These results suggest that our diagnostic model has a high value in the diagnosis of AD.

## 4 DISCUSSION

AD is a common neurodegenerative disease that has been widely studied globally. Although some progress has been made, the existing therapies are unsatisfactory because of the lack of adequate biomarkers and the heterogeneity of this disease. The identification of disulfidptosis‐related clusters in the present study provides novel molecular stratification methods for improving individualized therapeutic strategies for patients with AD. Moreover, the successful construction of a prediction model based on these molecular clusters could further assist in clinical decision‐making regarding AD diagnosis.

We conducted a comprehensive analysis of the expression patterns of DRGs between the AD and control samples. Eight of 10 DRGs showed differential expression and exerted significant synergistic or antagonistic effects, indicating the crucial function of DRGs in the development and progression of AD. We then performed an unsupervised clustering analysis to categorize the AD samples into various disulfidptosis‐related molecular clusters using the expression landscapes of the DRGs. Two separate disulfidptosis‐related clusters were identified. The GSVA results showed that cluster 1 was significantly associated with immune‐related pathways. Additionally, the CIBERSORT algorithm revealed significant differences in 12 of 22 types of immune cells between the AD and control samples, suggesting the potential role of immune cells in the development of AD. Mounting evidence suggests that the pathogenesis of AD is not confined to the neuronal compartment, but instead actively involves immune mechanisms in the brain.^37^, ^38^, ^39^, ^40^ When aggregated and misfolded proteins bind to pattern recognition receptors on microglia and astroglia, they induce an innate immune response that involves the release of inflammatory mediators that play key roles in disease severity.^41^, ^42^, ^43^, ^44^ This is consistent with the results of the immune cell infiltration analysis performed in the present study, which indicated that immune dysfunction was associated with AD. The classification of patients with AD into disulfidptosis‐related clusters revealed differences in multiple types of immune cells, implying that further research is necessary to explore possible differences in outcomes.

Nowadays, machine learning has exhibited excellent performance in clinical diagnosis; thus, it is widely applied to predict new biomarkers and provide novel insights into disease pathogenesis. In the present study, we used various machine‐learning algorithms to generate prediction models based on the hub genes identified by WGCNA on the training cohort and disulfidptosis‐related clusters. The results indicated that SVM yielded superior diagnostic outcomes for AD than the other algorithms. The SVM model showed the most favorable performance with the lowest residuals and highest AUC values. Therefore, the top five essential feature variables (*FXYD5*, *NRXN3*, *SERTAD3*, *AEBP1*, and *PAK1*) revealed by the SVM model were considered as the optimal signature gene panel. *PAK1* was one of the primary isoforms of PAK present in the brain, with a diffuse distribution across cell bodies and dendrites, and was implicated in the synaptic and cognitive dysfunctions in AD.^45^ *NRXN3* exhibited distinct essential pre‐ or postsynaptic functions in various brain regions and its dysregulation in presynaptic expression and splicing might contribute to increased neuronal inflammation in the brain of patients with AD.^46^, ^47^, ^48^ *AEBP1* plays a role in the progression of AD pathology.^49^, ^50^, ^51^ Moreover, *FXYD5* can downregulate E‐cadherin and promote metastasis,^52^ whereas *SERTAD3* is a strong transcriptional co‐activator with high activity.^53^ The precise functions of *FXYD5* and *SERTAD3* in AD are yet to be fully elucidated, and our findings offer new insights into AD pathogenesis. Additionally, the nomogram based on this gene model exhibited remarkable accuracy in predicting AD. Importantly, five external datasets were included to validate the developed gene model, and the robustness of the model was confirmed by AUC values consistently exceeding 0.75. Thus, our findings provide compelling evidence that the established model is a dependable tool for AD diagnosis.

Nevertheless, the study has some limitations. We used multiple datasets extracted from the GEO database, so necessitating prospective investigations are needed to determine the efficacy of the model when applied to clinical samples. Additionally, the molecular mechanisms underlying the predictive model were not elucidated or validated via experimental studies. Furthermore, additional samples are necessary to clarify the accuracy of the disulfidptosis‐related clusters and the correlation between DRGs and immune responses in AD. We plan to address these limitations in future research.

In conclusion, we demonstrated substantial heterogeneity in immune cell populations among AD patients with different disulfidptosis‐related clusters. A five‐gene‐based SVM model was selected as the optimal model for predicting AD. The AUC values were consistently above 0.75 in the training and validation cohorts, suggesting the reliability of the diagnostic prediction model and the possibility of its effective integration into clinical practice. These findings have significant clinical implications regarding the role of disulfidptosis in AD heterogeneity and the development of targeted therapies for individuals with AD.

## AUTHOR CONTRIBUTIONS

**Yidong Zhu**: Conceptualization (lead); writing—original draft (equal); formal analysis (lead). **Lingyue Kong**: Review and editing (equal). **Tianxiong Han**: Review and editing (equal). **Qiongzhi Yan**: Original draft (equal); review and editing (equal). **Jun. Liu**: Writing—review and editing (equal). All read and authors gave their approval for publication of the final version of the manuscript.

## CONFLICT OF INTEREST STATEMENT

The authors declare no conflict of interest.

## Supporting information

Supporting information.

## Data Availability

The datasets used and/or analyzed in the current study are available from the corresponding author upon reasonable request.
