# Supplementary material for: Machine learning identification and immune infiltration of disulfidptosis‐related Alzheimer's disease molecular subtypes
Source: Immun Inflamm Dis. 2023 Oct 11;11(10):e1037. doi: 10.1002/iid3.1037 (PMC10566450; doi:10.1002/iid3.1037)
Supplement: Supplementary file 1 — Supporting information. [file IID3-11-e1037-s001.doc]

**Supplementary Materials**

**Supplementary Table S1** Disulfidptosis-related genes.

| **Disulfidptosis-related Genes** |
| --- |
| SLC7A11 |
| SLC3A2 |
| RPN1 |
| OXSM |
| NUBPL |
| NDUFS1 |
| NDUFA11 |
| NCKAP1 |
| LRPPRC |
| GYS1 |


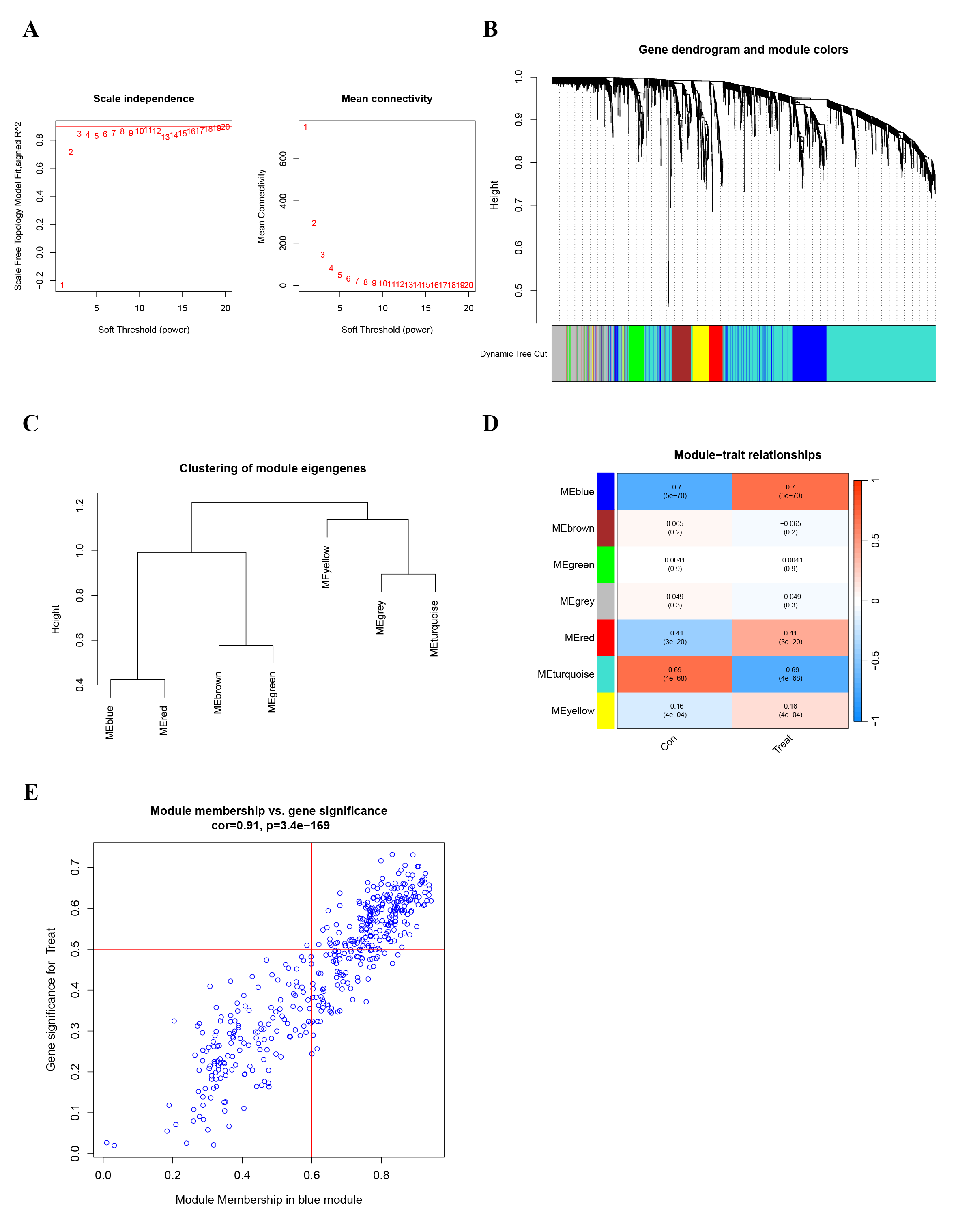


**Supplementary Figure 1. Construction of a gene co-expression network between AD and control samples.** (A) Analysis of the scale-free network topology for the optimal soft threshold; (B) gene dendrogram and module colors; (C) clustering of module eigengenes; (D) heatmap of the module-trait relationships; (E) scatter plot of module membership and gene significance in the blue module.





**Supplementary Figure 2. Construction of a gene co-expression network between the disulfidptosis-related clusters.** (A) Analysis of the scale-free network topology for the optimal soft threshold; (B) gene dendrogram and module colors; (C) clustering of module eigengenes; (D) heatmap of the module-trait relationships; (E) scatter plot of module membership and gene significance in the blue module; (F) Venn diagram of the AD-related genes from the training cohort and the cluster-specific genes from the disulfidptosis-related clusters.
